# The experiences and psychological impact of living with premenstrual disorders: a systematic review and thematic synthesis

**DOI:** 10.3389/fpsyt.2024.1440690

**Published:** 2024-09-02

**Authors:** Danielle Brown, Debbie M. Smith, Elizabeth Osborn, Anja Wittkowski

**Affiliations:** ^1^ Division of Psychology and Mental Health, Faculty of Biology, Medicine and Health, University of Manchester, Manchester, United Kingdom; ^2^ Psychology Services, Greater Manchester Mental Health National Health Service (NHS) Foundation Trust, Manchester, United Kingdom; ^3^ Paediatric Psychology Department, Glan Clwyd Hospital, Betsi Cadwaladr University Health Board, Rhyl, United Kingdom; ^4^ Manchester Academic Health Science Centre, Manchester, United Kingdom

**Keywords:** premenstrual syndrome, PMS, premenstrual dysphoric disorder, PMDD, women’s health, menstrual cycle

## Abstract

**Introduction:**

As the psychological impact and decreased quality of life experienced by women living with a Premenstrual Disorder (PMD) has been reported in the literature, the aim of this systematic review and thematic synthesis was to explore a) their experiences and the psychological impact of PMDs, specifically Premenstrual Syndrome (PMS) and Premenstrual Dysphoric Disorder (PMDD), and b) their perceived support needs.

**Method:**

Six databases were searched for publications reporting on qualitative studies, since the database inception. The Preferred Reporting Items for Systematic Review and Meta-Analysis guidelines were followed.

**Results:**

Seventeen papers reporting on 479 women met the inclusion criteria: ten focused on PMS, six on PMDD and one on PMS and PMDD combined. Two main PMD themes were identified: 1) *controlled by PMDs*, which had three subthemes, and 2) *a women and life left broken*, with five subthemes.

**Conclusion:**

Women’s accounts revealed that experiences of PMDs were intense, life changing and life-controlling. Women were left holding the responsibility of understanding and managing their own condition, whilst advocating for themselves in a healthcare setting in which their condition has been little understood. Consequently, women developed coping strategies to lead a functional life, and experienced changes to their sense of self. Clinical recommendations included the need for professionals working with women in crisis, to assess for PMDs and signpost towards specialist services.

## 1 Introduction

Premenstrual disorders (PMDs) are on a continuum of premenstrual symptoms ranging in severity from Premenstrual Syndrome (PMS) to the more debilitating Premenstrual Dysphoric Disorder (PMDD) (1), despite being diagnosed separately since 1987 (2). Given this continuum, both PMDD and PMS papers will be included within this review, under the term PMD. Up to 80% of women experience premenstrual symptoms each month (3), for approximately 20-40% of menstruating women these symptoms meet a clinically significant level, affecting their daily functioning, and are defined as PMS (2–4). Only 3-8% suffer symptoms severe enough to be classified as PMDD (2); however, prevalence rates vary depending on assessment method (5). At present, there is no clear understanding of the etiology of PMDs; however, theories include genetics, increased sensitivity of the central nervous system to menstrual cycle hormones and psychosocial factors [for a comprehensive overview, see Hantsoo and Epperson (6)].

Premenstrual Disorders are defined by the cyclical nature of their symptoms, occurring during the luteal phase and subsiding with menstruation, with a symptom-free period between menstruation and ovulation (2). Symptoms of PMDs include low mood, affective liability, and interpersonal conflicts, as well as physical discomfort, changes to appetite and sleep. According to the DSM-V (7), symptoms must cause an impairment to the individual’s daily personal, professional, or social commitments during the luteal phase to meet the threshold for a PMDD diagnosis. PMDD is linked to co-morbidities with depression, anxiety and panic disorders, as well as social phobia, OCD (8) and suicidal ideation (9).

Treatment options for PMDs are limited, and a cure for PMDD specifically is only truly possible by removing the ovaries (2). However, an individual’s day-to-day life can be improved through symptom management, such as prescribing antidepressants or hormone therapies, to reduce the fluctuation of hormone levels (2). For more mild symptoms, non-pharmacological treatment recommendations include cognitive behaviour therapy, dietary intervention, exercise, exposure to sunlight, stop smoking and not drinking alcohol (10).

In terms of interventions, Kancheva Landolt and Ivanov’s (11) systematic review of 32 peer-reviewed papers found non-pharmacological interventions provided a significant reduction in PMS symptoms. In addition, Carlini et al.’s (12) scoping review of 113 studies highlighted that PMS and PMDD symptom reduction was possible with both pharmacological and non-pharmacological interventions, but the authors expressed concern about the quality and methods of some non-pharmacology studies.

The impact of PMDs on a woman’s life has been documented by various quantitative studies (13), and although some women experienced their premenstrual changes positively (14), most literature recognises the negative impact. Experiencing PMDs placed a burden on women’s occupation (15) and daily activities (16), and has been associated with depression, stress, sleep disturbances and a poor relationship with food (17). Prabhavathi et al. (18) found that as the severity of PMS symptoms increased, cognition and psychomotor execution decreased, highlighting the impact symptoms had on a woman’s functional abilities. Given the vast impact of PMDs, it is unsurprising that data from 500 female students showed a direct association with PMS and decreased quality of life measures (19).

In Osborn et al.’s (20) review of ten quantitative studies, women with PMDD were noted to be a high-risk group for suicidal ideation; however, the authors did not find women with PMDD to be at a higher risk for suicide attempts. In contrast, Prasad et al.’s (9) review of 13 papers identified an almost sevenfold increase in risk of suicide attempts. Finally, in the only review of the qualitative literature to date, Moe and Karlsson (21) identified 12 papers reporting on the experiences of women with PMDD only. Two main themes identified the social, emotional, and professional limitations women experienced due to PMDD and their journey to a diagnosis and treatment options. Although the authors used a comprehensive approach to provide nursing specific clinical recommendations, they did not explore the psychological impact of this particular diagnosis, nor did they highlight how services could support these women.

There is a growing qualitative literature exploring women’s experiences of PMDs. Changes to women’s body dissatisfaction have been documented across the menstrual cycle, and many women chose to conceal their body during the premenstrual phase (22). Cosgrove and Riddle (23) interviewed 30 women with PMS and described the contrast between women’s view of themselves with and without their symptoms, leading them to question which was their true identity. Uncertainty about one’s own self could be connected to women’s reported feelings of loneliness (24). These studies provide insight into the affect PMDD has on a woman’s self-image and identity but lacked a comprehensive exploration of the wider psychological impact. As previously discussed, PMDs are considered to sit on a continuum of symptom severity (1), as recognised by Carlini et al. (12) in their review of interventions. As a synthesis of PMDs experiences could provide novel insights into their psychological impact on women. Therefore, the proposed review of qualitative studies aimed to a) explore women’s lived experiences of a PMD’s, such as PMS or PMDD, and b) explore their perceived support needs from healthcare services.

## 2 Method

### 2.1 Search strategy

The systematic search was conducted in line with the Preferred Reporting Items for Systematic Reviews and Meta-Analysis (PRISMA) guidelines (25) and the protocol was registered with PROSPERO in January 2024 (CRD42024505284). The SPIDER tool (26) categories phenomenon of interest (PI), design (D), and research type (R) were used to create search terms (see **Table 1**). Medical Subject Heading (MeSH) terms identified synonyms, whilst search categories were combined with Boolean operator “AND”. Due to diagnostic terminology changing from LLPDD to PMDD in the DSM-IV in 2000 (7, 27), the decision was made to include PMD, PMS, PMDD and LLPDD within the search terms, to ensure no eligible papers were omitted. Six databases were searched from inception to March 2024, CINAHL (EBSCO), EMBASE (OVID), HMIC (OVID), Medline (OVID), PsycINFO (OVID) and Web of Science. Backwards searching of identified papers’ reference lists and papers citing the included papers were also used.

**Table 1:** Search terms by category and search strategy.

	Search terms	
1	(PI) Phenomenon of interest	“Premenstrual dysphoric disorder*” OR PMDD OR “premenstrual syndrome*” OR PMS OR “late luteal phase dysphoric disorder*” OR LLPDD OR “premenstrual disorder*” OR PMD
2	(D) Design	Interview* OR “focus group*” OR questionnaire* OR survey* OR “case stud*”
3	(R) Research Type	Qualitative* OR “mixed method*”
4	2 OR 3	
5	1 AND 4	

### 2.2 Inclusion and exclusion criteria

Papers were included if 1) participants experienced PMS or PMDD, with a self-reported diagnosis or diagnosis confirmed by study or medical team, 2) studies aimed to understand the participants’ experiences related to their condition, 3) studies which utilised qualitative research methods for data collection and analysis (e.g., interviews), including mixed method studies in which qualitative results were presented separately, and 4) studies written or translated into English. Papers were excluded if 1) participant eligibility was unclear or their diagnosis was vague, 2) participants with and without a diagnosis were recruited, and without findings reported separately, or 3) they reported on secondary research (e.g., conference posters or literature reviews).

### 2.3 Quality appraisal

The Critical Appraisal Skills Programme (28) tool is a validated checklist used to assess included papers, with ten domains including methodology, ethical issues and results. As the CASP does not offer a summary scoring system (29), a numerical system was also used for better comparison across reviews (yes=1, partially agree=0.5, no=0). Total CASP scores were used to categorise methodological quality as high (> 8-10), moderate (6-8) or low (<5) (30, 31). As no accepted guidelines for excluding studies based on quality exist (32, 33), all studies were included irrespective of quality appraisal.

### 2.4 Data extraction and data analysis

All eligible papers were transferred into NVivo software in preparation for analysis and relevant study characteristics (e.g., aims, sample size and recruitment strategy) were extracted and tabulated. Thematic synthesis (32) was used for data analysis and involved three stages: line-by-line coding of the individual papers’ findings was completed independently by two of the authors (DB & DMS), codes were then grouped into descriptive themes across and between papers, with the reviewers looking for similarities and differences between the codes. All themes were discussed and finalised by the whole team, allowing different perspectives and judgements of the meaning behind each code.

### 2.5 Reflexivity statement

All authors were white women and mothers; however, they ranged in age and stage of their careers. The first author (DB) was a trainee clinical psychologist, with experience working with women in secure services and supporting children and families in community services. The second author (DMS) was a Health Psychologist and Senior Lecturer, specialising in exploring pregnancy and behaviour change. The third author (EO) was a Clinical Psychologist working in paediatric services and had an interest in premenstrual disorder research. The fourth author (AW) was a Clinical Psychologist and Senior Lecturer, with an interest in understanding mothers experiencing severe mental health difficulties. As a team, we acknowledged our similarities with the participants as females of reproductive age, whilst holding in mind the potential for power differentials between researchers without a premenstrual condition and participants with a diagnosis. The similarities and differences between the research team supported nuances in interpretation during the synthesis, whilst discussions and reflective diaries were utilised to minimise the risk of biased interpretations.

## 3 Results

### 3.1 Search outcome

**Figure 1:**
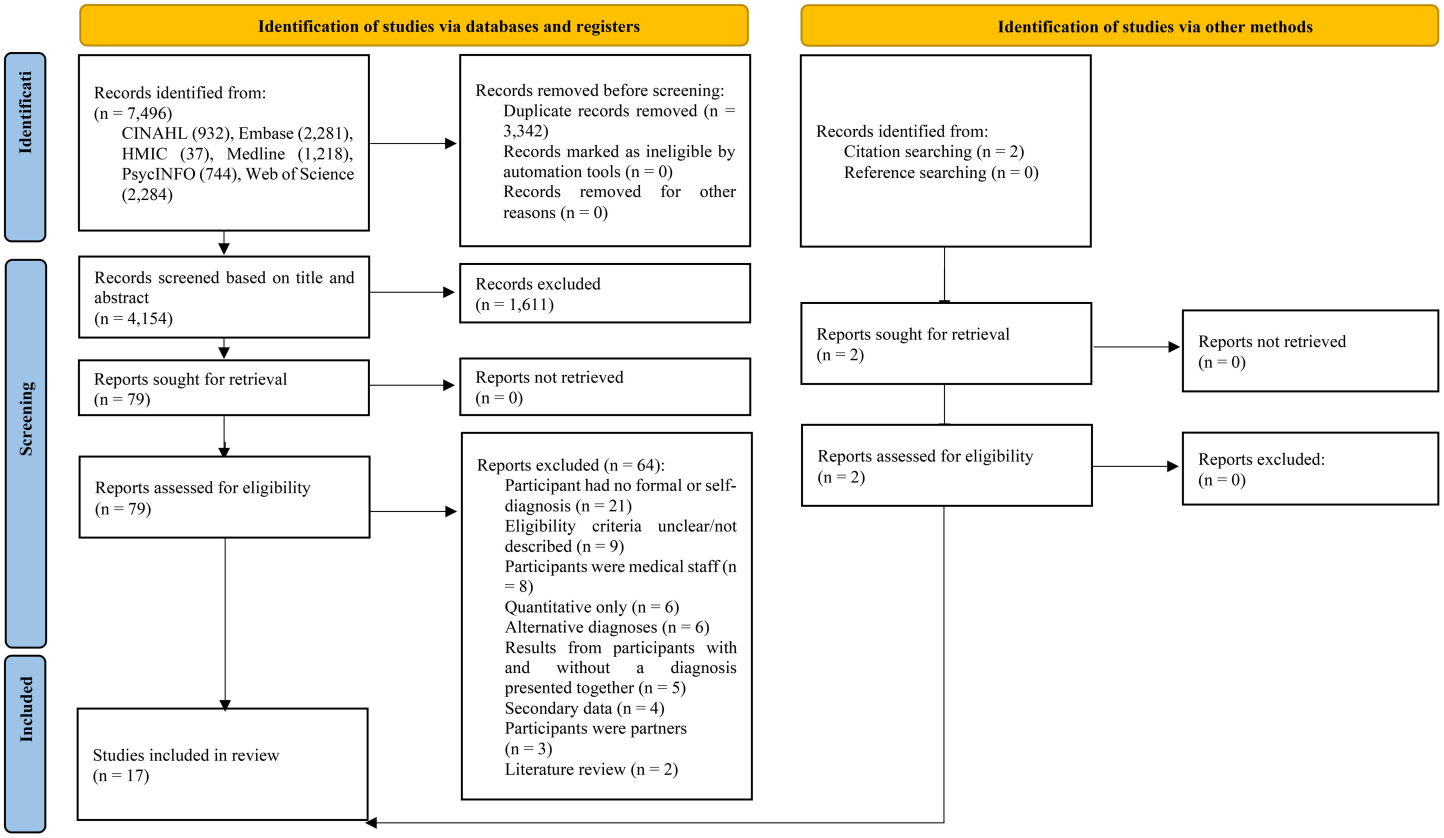
PRISMA diagram of the search strategy (25).

Initial searches identified 7,496 references. Following the removal of duplicates, the title and abstract of 4,154 papers were screened for eligibility (see **Figure 1**). The full text of 79 studies was assessed, with 15 selected for inclusion. An additional two papers were included following backwards searches of the citations and references, resulting in 17 included papers. An independent researcher (SH) assessed 10% of the search results against the eligibility criteria: there was a 100% agreement based on the title and abstract and 100% agreement after reading the full papers (kappa=1).

### 3.2 Characteristics of included studies

Seventeen papers, published from 1993 to 2024 and conducted in ten countries, were identified and synthesised (see **Table 2**). Sample sizes ranged from four to 83, with a combined sample of 479. Six studies recruited women with a diagnosis of PMDD: four studies allowed participants to self-report their formal diagnosis, one study confirmed diagnosis using the *Premenstrual Symptoms Screening Tool* (PSST) (40) and the final study stated that the participants met the DSM-IV criteria for PMDD. One publication used the International Society for Premenstrual Disorders definition of Premenstrual Disorders (PMDs; which includes PMDD and PMS) as eligibility for participation. The other ten studies recruited women with PMS: six accepted self-report diagnosis, two used the PSST, and two stated that researchers confirmed PMS symptoms or diagnosis (see **Table 2**). Women were recruited from a range of settings, including social media, local newspapers, and radio adverts, as well as medical clinics and snowball sampling. Fifteen studies collected data via semi-structured interviews, the remaining two studies used interviews as well as open-ended surveys and a questionnaire followed by case studies. Thematic analysis was the most used analysis method (n=9), two studies used thematic decomposition and two described content analysis. Thematic coding, narrative analysis, the listening guide and a feminist phenomenological approach were each referenced once.

**Table 2:** Characteristics of included studies.

	Author (date) [ref] Location	Aims	Sample size and diagnosis	Participant characteristics	Recruitment and data collection	Methodology and analysis	Findings and themes
PMDD only studies							
1	Buys (2024) (34) Australia	To explore the transition into recovery, management or transformation of PMDD and how participants understood those narratives	7 Self-diagnosed or reported a formal diagnosis of PMDD, but identified as in recovery, management, or transformation of PMDD	22-45 years old 57% Australian, 28% British, 15% Turkish 85% employed, 15% student Age at onset of symptoms, age at diagnosis and relationship status not reported	Social media adverts and online support groups. Narrative interviews	The Listening Guide) (35, 36)	Two narrative themes: 1) within abjection 2) beyond abjection
2	Chan et al. (2023) (37) USA	To explore the diagnostic and treatment experiences of PMDD patients in the U.S. healthcare system and identify barriers to diagnosis and treatment	32 Self-identified having PMDD (87% reported formal diagnosis)	21-50 years old Experienced PMDD symptoms for a mean of 17.43 years and mean of 5.6 years from symptom onset to diagnosis Age at diagnosis ranged from 16 to 45 94% white, 12% Hispanic, 3% Alaskan native, 3% mixed ethnicity 65% single, 29% married and 6% divorced 86% had attended collage	Online adverts (supported by IAPMD) Semi-structured interviews	Feminist phenomenological approach (38)	Study presents a PMDD Care Continuum that represents five themes as a timeline of participant experiences: 1) PMDD Symptoms 2) Patient delay 3) Diagnosis delay 4) Treatment delay 5) Condition management delay
3	Osborn et al. (2020) (39) England	To explore women’s experiences of both having PMDD and of receiving this diagnosis	17 PMDD diagnosis confirmed by the *Premenstrual Symptoms Screening Tool* (PSST) (40) questionnaire	20-56 years old Average symptom onset of 15 years old Average diagnosis of 35 years old 83% white British 47% married, 47% single, 6% divorced 53% obtained undergraduate degree or higher 59% mothers	Recruited via two NHS gynaecology clinics. Semi-structured interviews	Reflexive thematic analysis (41, 42)	Four themes: 1) A broken woman 2) Misdiagnosis and the lost decades 3) A life transformed 4) Negotiating the aftermath
4	Marfuah and Barat (2018) (43) Indonesia	To understand the experiences of adolescents with PMDD	6 Met the diagnostic criteria for PMDD with DSM – IV	14-18 years old Age of first menstruation was between 10 and 15years old 100% Javaness 100% Students relationship status not reported	Purposive sampling from one collage Interviews	Thematic analysis (no reference within paper)	Four themes: ***3) *** Symptoms perceived as a change that affects the psychological, behavioural and physical teens 2) Symptoms of intermittent throughout the menstrual cycle 3) Environmental factors and hormones play a role in the emergence of symptoms 4) The symptoms cause discomfort and interfere with social relationships
5	Hardy and Hardie (2017) (44) England	To explore women’s experience of PMDD in the workplace	15 Self-reported a formal diagnosis of PMDD	25-49 years old 80% receiving treatment for PMDD Received a diagnosis 6months to 4years prior to the interview 53% British, 40% American, 13% did not disclose 87% employed, 13% unemployed relationship status not reported	Online adverts via social media Semi-structured interviews	Thematic analysis (41)	Two themes: 1) Phases of PMDD at work and 2) The role of the organisation
6	Jurvanen (2017) (45) Sweden	To understand the subjective experiences of private- and work life for people with PMDD	11 Self-reported a formal diagnosis of PMDD	55% worked full time, 18% worked part time, 27% were freelance Age range, age at onset of symptoms, age at diagnosis, ethnicity and relationship status not reported	Social media adverts Semi-structured interviews	Thematic analysis (41)	Five themes: 1) The impact of PMDD on work and occupational life 2) PMDD and social life 3) Psychological welfare and PMDD 4) Medical shortcomings 5) Participants’ thoughts
PMDD and PMS studies							
7	Labots-Vogelesang et al. (2023) (46) Netherlands	To improve understanding of the perspectives of women with PMD, their coping strategies and their expectations of the GP	20 Researchers confirmed symptoms met IAPMD definition of PMD	27-49 years old PMD symptoms started at 14 to 43 years old 95% Dutch, 5% Moroccan 75% married/partnership, 20% single, 5% widowed 80% employed, 15% unemployed, 5% student 60% mothers	Adverts in local newspapers and closed PMS/PMDD Facebook pages. Semi-structured interviews	Thematic analysis (The qualitative data analysis & research software) (47)	Three themes: 1) Separate female identities 2) A life-controlling condition 3) Differences in coping strategies
PMS only studies							
8	Park et al. (2023) (48) England	What are the lived experiences of women with PMS? To what extent does PMS influence their daily occupations? What are the needs of women with PMS	4 Self-reported PMS symptoms	No participants details reported	Social media adverts. Semi-structured interviews	Inductive thematic analysis (41)	Three themes: 1) Occupational disturbance 2) Social impairment and occupational disengagement 3) The importance of self-awareness to engage in occupations
9	Tutty et al. (2022) (49) Canada	To explore the relationship between women’s premenstrual symptoms and parenting stress	46 Mothers who self-reported PMS	23-47 years old 72.7% white, 16.4% Indigenous, 3.6% Vietnamese, 3.6% Filipino, 3.6% East Indian 65.5% married or living with a partner, 34.5% lived alone Mothers of between 1 and 6 children, with at least one child under 18 80% attended post-secondary schooling 51% employed outside of the home, 37.7% not employed outside of the home, 11.3% students 27.2% currently taking anti-depressants Age at onset of symptoms and age at diagnosis not reported	Adverts placed in local newspapers and public locations, including; libraries, hospitals and child welfare offices. Mixed methods, semi-structured interviews	Thematic analysis (41)	Three themes: 1) Effects of PMS on mothering 2) Parenting changes after bad premenstrual phases 3) Strategies to address negative mothering
10	Ussher and Perz (2020) (50) Australia	To examine the role of premenstrual embodiment in women’s premenstrual distress	83 Self-reported PMS, symptoms assessed with the *PSST* (40) and daily diary measures	Average age 35 years old 100% in a relationship 98% were heterosexual, 2% lesbian Age at onset of symptoms, age at diagnosis and ethnicity not reported	Participants recruited from a larger scale project (51). Recruited via social media, local radio, newspapers and women’s health centres. Open-ended survey responses and interviews	Theoretical thematic analysis (41)	Two themes: 1) Inhabiting the abject premenstrual body 2) Reframing premenstrual embodiment: resisting the self-objectification and dehumanization.
11	Labots-Vogelesang et al. (2019) (52) Netherlands	To explore which symptoms/complaints are considered most disabling and why, what cognitions women have about the cause of PMS and how these affect their help-seeking behaviour	20 Women who met DSM-5 criteria for PMS, confirmed by researcher	27-49 years old PMS symptoms started at 14 to 43 years old 95% Dutch, 5% Moroccan 75% married/partnership, 20% single, 5% widowed 80% employed, 15% unemployed, 5% student 60% mothers	Recruited via local newspapers and social media Semi-structured interviews	Thematic coding (no reference) Consolidated Criteria for Reporting Qualitative Studies (COREQ) (53)	Three themes: 1) The disturbance in preferred feminine roles of being a good mother and wife 2) PMS as a life-controlling condition 3) Differences in coping strategies
12	Siahbazi et al. (2018) (54) Iran	To discover the experiences of women with PMS, with a focus on quality of life	21 Moderate to severe PMS based on the *PSST* (40)	15-45 years old 48% married, 48% single, 4% divorced 43% mothers (between 1 and 3) 58% employed, 28%, housekeeper 14% students 67% attended higher education, 33% high school education Age at onset of symptoms, age at diagnosis and ethnicity not reported	Purposive sampling Semi-structured interviews	Content analysis (55)	Four themes: 1) Physical consequences 2) Psychological consequences 3) Behavioural consequences 4) Familial-social consequences
13	Ussher and Perz (2013) (56) Australia	To identify key themes in women’s construction and experience of premenstrual change, and the ways in which women negotiate and cope with PMS, in the con- text of relationships	60 Self-reported to experience PMS	22-48 years old 98.5% Anglo-Australian, 1.5% Asian 80% in a relationship 66% heterosexual, 34% lesbian 47% mothers 82% employed Age at onset of symptoms and age at diagnosis not reported	Participants recruited from a larger scale project (50). Recruited via social media, local radio, newspapers and women’s health centres. Semi-structured interviews	Thematic analysis (41, 57)	Three themes: 1) Self-monitoring and awareness 2) Recognition and acceptance of premenstrual change 3) Coping through self-regulation of premenstrual distress
14	Hoga et al. (2010) Brazil (58)	To describe the perceptions of women with PMS regarding the behaviour of their spouses in face of this event	20 Self-report PMS symptoms	19-44 years old 55% single, 35% married and 10% divorced 95% employed Years in education 10-16years Age at onset of symptoms, age at diagnosis and ethnicity not reported	Snowball sampling Semi-structured interviews	Narrative analysis (59)	Three themes: 1) Difficulties in identifying the syndrome and in adopting care practices 2) Lack of knowledge and sensitivity of men 3) Its impact on the couple relationship
15	Mooney-Somers et al. (2008) (60) Australia	To examine the development, experience and construction of premenstrual symptoms across a range of relationship types and contexts	60 Self-reported PMS	22-48 years old Majority Anglo-Australian 80% partnered 66% heterosexual, 33% homosexual 47% mothers Age at onset of symptoms and age at diagnosis not reported	Mixed method Recruited via local media, women’s health centres, community groups and social organizations. Semi-structured interviews	Thematic decomposition (61), a version of thematic analysis (41)	Three themes: 1) Naming to explain 2) ‘PMS’ becoming the only explanation for distress 3) ‘PMS’ as not a legitimate explanation for distress
16	Perz and Ussher (2006) (62) Australia	To examine women’s subjective experience of PMS, and the negotiation of PMS in the context of relationships	35 (interviews) 2 (case studies) Self-reported to experience PMS	17-49 years old 63% partnered 59.5% heterosexual 76.6% employed 44% mothers Age at onset of symptoms, age at diagnosis and ethnicity not reported	Mixed methods Recruited via local media and women’s health centres Questionnaire, narrative interviews, followed by case studies	Thematic decomposition (61)	Women described PMS similarly, as being characterized by intolerance, irritation, emotional sensitivity, feeling more negative towards others, and feeling overwhelmed in the face of life’s demands.
17	Burrage and Schomer (1993) (63) South Africa	To examine whether the daily coping processes of women suffering from PMS vary across the menstrual cycle and to investigate the effect that women’s coping resources have on the severity of their premenstrual symptom	12 PMS symptoms confirmed by researcher	30-49 years old 83% married, 17% single 33% housewives, 25% secretaries, 8% nurse, 8% musician, 8% part-time worker 8% used oral contraception Age at onset of symptoms, age at diagnosis and ethnicity not reported	Recruited via advert in local newspaper Women completed 8 weeks of daily PMS symptom diaries Three formal semi-structured interviews	Content analysis (64)	Interview 1: common PMS experiences and feeling like two different people. Interview 2: difficulty in meeting daily demands and interpersonal conflict. Interview 3: hassles arising from family matters, role conflict and daily workload.

### 3.3 Methodological quality of included studies

The methodological quality of all 17 studies was assessed as high (n=14) or moderate (n=3), indicating the rigorous analysis and reporting of results presented. Only three studies had sufficiently considered the researcher-participant relationship (39, 45, 48); however, all papers provided a clear statement of findings and described the value provided by their results. The CASP quality appraisal ratings can be viewed in **Table 3**. An independent researcher (SH) independently assessed all papers, there was a substantial agreement (97.3%, kappa=0.74), any discrepancies were resolved through discussion.

**Table 3:** Overview of CASP scores.

Author (year)		1. Clear Aims	2. Qual method appropriate	3. Research design appropriate	4. Recruitment strategy	5. Data collection	6. Researcher participant relationship	7. Ethical	8. Data analysis	9. Statement of findings	10. Value of research	Quality Score
PMDD only studies												
**1**	**Buys (2024)** (34)	Yes (1)	Yes (1)	Yes (1)	Yes (1)	Yes (1)	No (0)	No (1)	Yes (1)	Yes (1)	Yes (1)	High (9)
**2**	**Chan et al. (2023)** (37)	Yes (1)	Yes (1)	Yes (1)	Yes (1)	Yes (1)	No (0)	Yes (1)	Yes (1)	Yes (1)	Yes (1)	High (9)
**3**	**Osborn et al. (2020)** (39)	Yes (1)	Yes (1)	Yes (1)	Yes (1)	Yes (1)	Yes (1)	Yes (1)	Yes (1)	Yes (1)	Yes (1)	High (10)
**4**	**Marfuah and Barat (2018)** (43)	Yes (1)	Yes (1)	No (0)	Yes (1)	Yes (1)	No (0)	No (0)	No (1)	Yes (1)	Yes (1)	Moderate (6)
**5**	**Hardy and Hardie (2017)** (44)	Yes (1)	Yes (1)	Yes (1)	Yes (1)	Yes (1)	No (0)	Yes (1)	Yes (1)	Yes (1)	Yes (1)	High (9)
**6**	**Jurvanen (2017)** (45)	Yes (1)	Yes (1)	Yes (1)	Yes (1)	Yes (1)	Yes (1)	Yes (1)	Yes (1)	Yes (1)	Yes (1)	High (10)
PMDD and PMS studies												
**7**	**Labots-Vogelesang et al. (2023)** (46)	Yes (1)	Yes (1)	Yes (1)	Yes (1)	Yes (1)	No (0)	Yes (1)	Yes (1)	Yes (1)	Yes (1)	High (9)
PMS only studies												
**8**	**Park et al. (2023)** (48)	Yes (1)	Yes (1)	Yes (1)	Yes (1)	Yes (1)	Yes (1)	Yes (1)	Yes (1)	Yes (1)	Yes (1)	High (10)
**9**	**Tutty et al. (2022)** (49)	Yes (1)	Yes (1)	Yes (1)	Yes (1)	Yes (1)	No (0)	Yes (1)	Yes (1)	Yes (1)	Yes (1)	High (9)
**10**	**Ussher and Perz (2020)** (50)	Yes (1)	Yes (1)	No (0)	Yes (1)	Yes (1)	No (0)	No (0)	Yes (1)	Yes (1)	Yes (1)	Moderate (7)
**11**	**Labots-Vogelesang et al. (2019)** (52)	Yes (1)	Yes (1)	No (0)	Yes (1)	Yes (1)	No (0)	Yes (1)	Yes (1)	Yes (1)	Yes (1)	High (8)
**12**	**Siahbazi et al. (2018)** (54)	Yes (1)	Yes (1)	Yes (1)	No (0)	Yes (1)	No (0)	Yes (1)	Yes (1)	Yes (1)	Yes (1)	High (8)
**13**	**Ussher and Perz (2013)** (56)	Yes (1)	Yes (1)	Yes (1)	Yes (1)	Yes (1)	No (0)	Yes (1)	Yes (1)	Yes (1)	Yes (1)	High (9)
**14**	**Hoga et al. (2010)** (58)	Yes (1)	Yes (1)	Yes (1)	Yes (1)	Yes (1)	No (0)	Yes (1)	Yes (1)	Yes (1)	Yes (1)	High (9)
**15**	**Mooney-Somers et al. (2008)** (60)	Yes (1)	Yes (1)	No (0)	Yes (1)	Yes (1)	No (0)	Yes (1)	Yes (1)	Yes (1)	Yes (1)	High (8)
**16**	**Perz and Ussher (2006)** (62)	Yes (1)	Yes (1)	Yes (1)	Yes (1)	Yes (1)	No (0)	Yes (1)	Yes (1)	Yes (1)	Yes (1)	High (9)
**17**	**Burrage and Schomer (1993)** (63)	Yes (1)	Yes (1)	No (0)	Yes (1)	Yes (1)	No (0)	No (1)	Yes (1)	Yes (1)	Yes (1)	Moderate (7)
	Percentage of studies rated ‘Yes’ (1)	100%	100%	70%	94%	100%	18%	88%	94%	100%	100%	

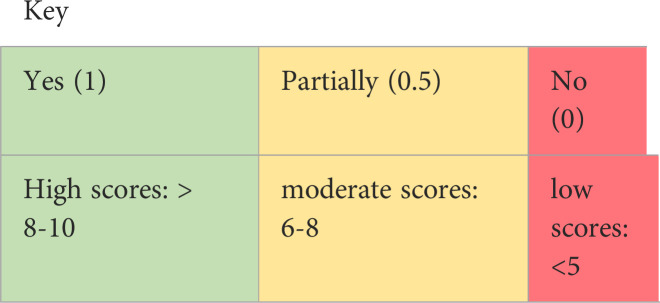

### 3.4 Thematic synthesis

**Figure 2:**
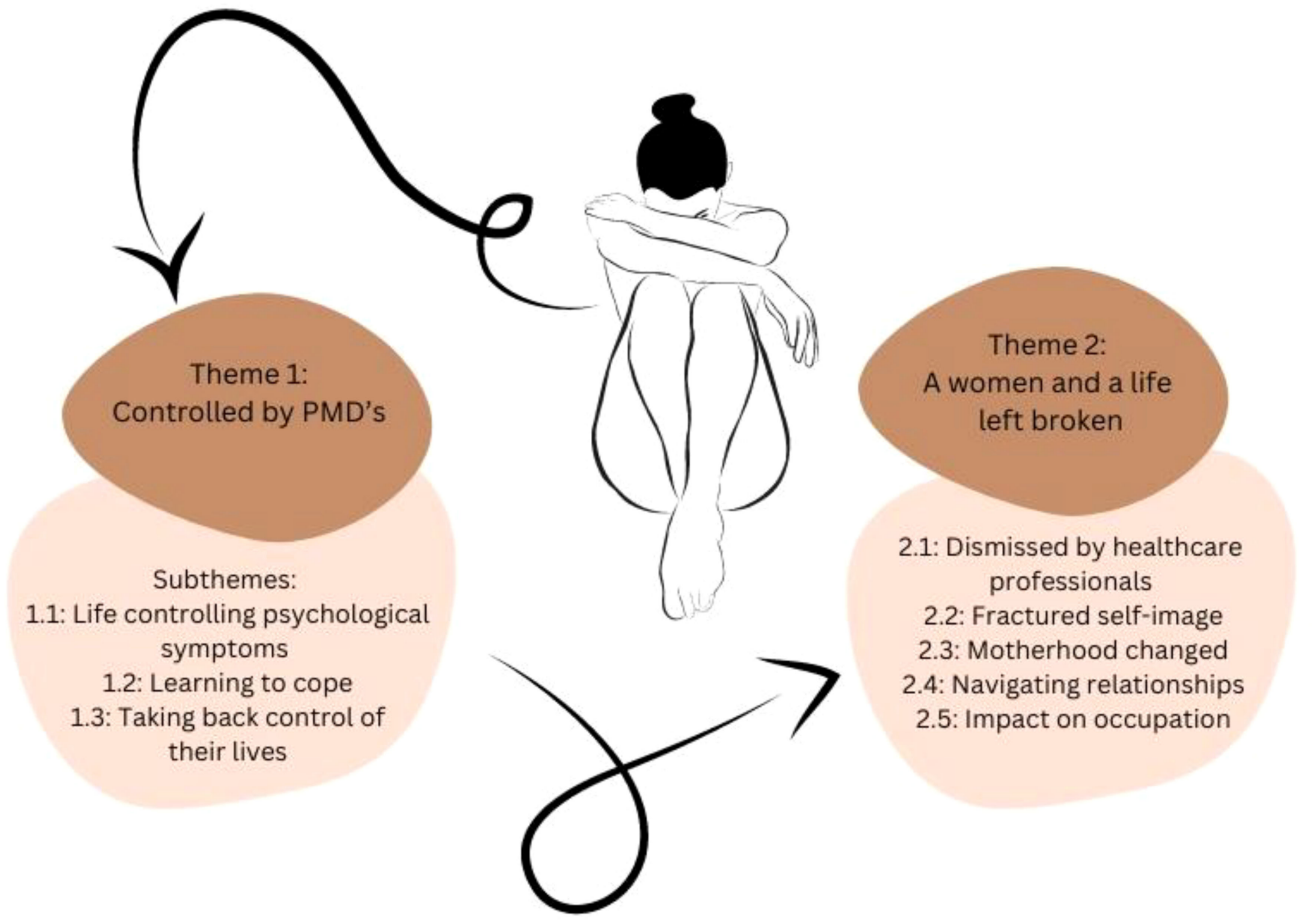
Overview of the two themes and subthemes.

Two main themes were conceptualized to capture women’s experiences. PMDs were described as life controlling, narratives indicated the psychological symptoms and maladaptive coping mechanisms left women feeling themselves and their lives were broken, and forever damaged. The two themes were 1) *controlled by PMDs* (with three subthemes) and 2) *a woman and a life left broken* (with five subthemes) (see **Figure 2**).

Themes are outlined below with their respective subthemes and quotes to support. **Table 4** presents a matrix of these themes and their respective subthemes, highlighting which themes were endorsed by each of the 17 studies.

**Table 4:** Matrix of theme representation within the included 17 studies.

Author (year)		Theme 1: Controlled by PMS/PMDD			Theme 2: A woman left broken				
		Life controlling psychological symptoms	Learning to cope	Taking back control	Dismissed by healthcare professionals	Fractured self-image	Motherhood changed	Navigating relationships	Impact on occupation
PMDD only studies									
1	Buys (2024) (34)	✓	✓	✓	✓	✓	✓	✓	✓
2	Chan et al. (2023) (37)	✓	✓	✓	✓	–	–	–	–
3	Osborn et al. (2020) (39)	✓	✓	✓	✓	✓	✓	✓	✓
4	Marfuah and Barat (2018) (43)	✓	✓	–	–	–	–	✓	✓
5	Hardy and Hardie (2017) (44)	✓	✓	✓	✓	✓	–	–	✓
6	Jurvanen (2017) (45)	✓	–	–	–	✓	✓	–	✓
PMDD and PMS studies									
7	Labots-Vogelesang et al. (2023) (46)	✓	✓	✓	✓	✓	✓	✓	✓
PMS only studies									
8	Park et al. (2023) (48)	✓	✓	–	✓	✓	✓	✓	✓
9	Tutty et al. (2022) (49)	✓	✓	–	✓	–	✓	✓	–
10	Ussher and Perz (2020) (50)	✓	✓	–	–	✓	–	✓	–
11	Labots-Vogelesang et al. (2019) (52)	✓	✓	–	✓	✓	✓	✓	–
12	Siahbazi et al. (2018) (54)	✓	–	–	✓	✓	✓	✓	✓
13	Ussher and Perz (2013) (56)	✓	✓	–	–	✓	✓	✓	✓
14	Hoga et al. (2010) (58)	✓	✓	–	–	–	–	✓	–
15	Mooney-Somers et al. (2008) (60)	✓	✓	–	–	✓	–	✓	–
16	Perz and Ussher (2006) (62)	✓	✓	–	–	✓	–	✓	✓
17	Burrage and Schomer (1993) (63)	✓	✓	–	✓	✓	✓	–	✓

#### 3.4.1 Theme 1: controlled by PMDs

This theme and its three subthemes captured the perceived control that PMDs exerted over women’s lives, and the coping strategies women developed as a result, including active strategies and avoidance. The process of regaining control was framed as separately to coping strategies implemented, and therefore described as a separate subtheme.

##### 3.4.1.1 Subtheme 1.1: life-controlling psychological symptoms

Psychological and behavioural symptoms of both PMS and PMDD were reported as negatively influencing quality of life more than any physical symptoms. The psychological impacts were defined as “*life-controlling*” [ (52): p.5], with examples including emotional sensitivity, feeling overwhelmed and negatively towards others. At their most extreme, women reported suicidal thoughts and attempts to end their life and “*monthly admissions to emergency department each time they reached crisis point*” [ (39): p.7]. For some women, the time without symptoms was spent preparing for and worrying about their next premenstrual phase, highlighting the life-controlling nature of the condition.

“*I’m actually always thinking about it. And when I feel good, I’m already preoccupied with it, like: ‘Oh, I hope I won’t feel bad again*’” [ (6): p.5].

##### 3.4.1.2 Subtheme 1.2: learning to cope

A wide variety of coping strategies to manage the symptoms and impacts of their undiagnosed PMDs were described, ranging from active approaches to avoidance. Although many papers referenced isolation, there was an interesting contrast in framing: some describing avoidance of “*emotional labour*” [ (62): p.297], whilst others reported being alone as a form of self-care.

“*I just want to lock myself in a room and hide under a duvet and not talk to or see anyone. And I’m completely disengaged and don’t take initiatives*” [ (45): p.25].

Many women had developed maladaptive coping strategies; for example, substance misuse, self-harm or disordered eating as a way to maintain control or as a form of self-harm. Secondary mental health difficulties were also described; eating disorders and suicidal thoughts or attempts to end their lives.

“*And so at some point I [ … ] would also feel the urge to end it all*” [ (46): p.5].

Whilst some women lacked the energy to implement any coping strategies, others actively engaged with activities to look after their own body and prioritise themselves; “*taking the time-out to recognize my own needs has been very useful*” [ (50): p.15].

##### 3.4.1.3 Subtheme 1.3: taking back control of their lives

Shared amongst some participants was the sense of women taking back control of their lives, in contrast to feeling controlled by their PMDs, after receiving a diagnosis and/or treatment. This subtheme was more prevalent within the PMDD papers (see **Table 4**). Examples included women *“adjusting [their] lifestyle completely”* [ (34): p.11] and the ability to plan their lives around their menstrual cycles, rather than work against it. Although some women struggled to accept their diagnosis and were reluctant to take medications, others described validation from finally being given a diagnosis and/or treatment. Participants described their treatment as “*life changing and life-saving*” [ (39): p.8].

#### 3.4.2 Theme 2: a woman and a life left broken

Women described the length of time between their first symptoms and their eventual diagnosis, and the responsibility they held to advocate for themselves throughout this process. Advocating for themselves across a significant length of time when feeling repeatedly dismissed by healthcare impacted women’s sense of self, and other key life domains. Five subthemes were developed.

##### 3.4.2.1 Subtheme 2.1: dismissed by healthcare professionals

On many occasions women visited healthcare professionals seeking advice and help, but they left feeling “*dismissed*” [(39): p.7], with one individual being told her symptoms were “*in their head*” [ (37): p.4]. Professionals were deemed to have minimal knowledge regarding the symptoms or treatment options for PMDs, thus requiring women to be the expert and advocate for themselves.

“*I realised that I basically have to treat myself*” [ (48): p.643].

Women described spending months completing symptom diaries only to have doctors decline to read them, which they experienced as particularly frustrating because the DSM-V specifically highlights symptom diaries as a necessary part of the diagnostic process (7). If treatments were offered, these focused solely on physical symptoms, therefore not targeting most distressing psychological symptoms (as per theme 1, subtheme 1).

“*You can no longer turn to a doctor because [ … ] they had no answers either*” [ (46): p.5].

##### 3.4.2.2 Subtheme 2.2: fractured sense of self

Across the majority of studies, women used an array of terms to define and differentiate themselves with and without symptoms, as if they were two separate entities and described “*feeling like two different people*” [ (63): p.113]. Examples included “*alter-ego*” [ (39): p.5] and “*Jekyll and Hyde*” [ (44): p.294]. This finding appeared to be in response to the guilt and fear women experienced regarding their “*out of character*” [ (44): p.295] behaviours, whilst in the luteal phase. One consequence of a fractured self-image was a decline in self-esteem and self-confidence.

“*I lost my confidence and I stopped saying what I really felt and what I really thought*” [ (34): p.8].

Specifically, women described a self-objectification and annihilation of their “*sense of being attractive*” [ (50): p.7] and used derogatory terminology to describe themselves: *“frumpy”, “disgusting”* and *“unattractive*” [ (50): p.7]. Women chose to conceal their body during their premenstrual phase by wearing looser fitting clothes, or by simply not leaving the house.

##### 3.4.2.3 Subtheme 2.3: motherhood changed

For participants who had children, a majority spoke about the distress and impact of their symptoms on their children, and recognised being “*quite unreasonable with them*” [ (49): p.90] during their luteal phase. Women described the difficulties fulfilling their role as a mother and the impact this had; “*I feel like I’m not being a good mom*” [ (48): p.643]. Some women felt dominated by the needs of their family and described a feeling of resentment and internal conflict. Some women bravely shared their guilt of using physical chastisement with their children, such as spanking, when experiencing symptoms, something they would not typically do. Intense feelings of guilt then followed, and women overcompensated with their children afterwards or choose to isolate themselves during their luteal phase to avoid contact with their family to protect them.

“*For women who were mothers, they talked about having felt unable to care for their children and their deep regret for not having been able to be the parent that they wished to have been*” [ (39): p.7].

##### 3.4.2.4 Subtheme 2.4: navigating relationships

Maintaining relationships through menstrual cycles was a common challenge, women spoke of volatile relationships and repeated conflicts with partners, and experienced guilt for not fulfilling their own role as a supportive partner. Many spoke about their difficulties in having to rely on someone else for support and recognised the responsibility of having to educate their partner. Some women had a perception that their male partners did not understand their intense symptoms and they had a “*perception that men did not understand the suffering of women*” [ (58): p.375]. Relationships became fractured as partners told women that they “*cannot rely on you*” [ (54): p.288], resulting in women having to apologise for their behaviour during their luteal phase. However, when partners did recognise the difficulties, women generally felt more understood and supported. This perceived lack of understanding resulted in some heterosexual women never choosing to tell their partner when they were experiencing symptoms related to their menstrual cycle.

“*Very unfair that every month I have to say to my partner ‘no I’m, it’s the week that I’m getting my bad days so, you know, I’m just telling you now’ it’s a bit embarrassing*” [ (60): p.6].

##### 3.4.2.5 Subtheme 2.5: impact on occupation

As participants were working or were in education the term occupation was used to cover both activities. A common theme across studies was that maintaining an occupation whilst experiencing life-controlling symptoms was perceived to be a near impossible task. Women described regular absences, terminated employments and withdrawal from higher education: “*school was shattered*” [ (43): p.223].

“*I feel I can’t do the 8 hours a day, 5 days a week job. I really don’t think I could manage that mentally or physically. Because, if I look back at times I’ve been working, I have many days of absence. At least 2-3 days every month, and they always happen the days before menstruation*” [ (45): p.23].

Some women described feeling less motivation to engage in occupations during their luteal phase, whilst others acknowledged their careers had been impacted by their symptoms of emotional dysregulation.

“*Women often thought colleagues were talking about them and perceived them as being unable to do their job. Communications could often be misperceived as negative or a personal attack on them*” [ (44): p.294].

Avoidance was used by some women to manage at work, as well as recognising they held a more negative view of colleagues; “*I find I get more annoyed by other people … especially at work*” [ (50): p.915]. Although some individuals felt comfortable sharing their experiences with their employer, this came with its own complexities, including facing disciplinary action and justifying the chronic impact of PMDs.

## 4 Discussion

This systemic review of 17 studies was the first to explore and thereby report on the psychological impact of living with PMDs. Key themes highlighted PMS and PMDD were experienced as life controlling, women felt required to repeatedly advocate for themselves during appointments with medical professionals who failed to recognise their PMD, and they had to explain their condition to their family and work colleagues, who did not understand their symptoms’ psychological impact. The weight of this responsibility was with women who already experienced debilitating symptoms each month, which reduced their psychological resilience. Women positioned themselves as the expert, researching and educating others, including medical professionals. As a result of these demands, combined with living with life-controlling symptoms and developing and learning coping strategies to lead a functional life, women viewed themselves and their lives as broken.

The current review expands upon the findings of Moe and Karlsson’s PMDD review (21), the findings from both reviews support the impact PMD’s had on a variety of life domains, including family, relationships, and occupation. However, novel insights were provided by the current review into the relationships women held with others. Themes described the difficulties women had fulfilling their roles as a mother and partner, and the subsequent guilt and regret experienced. Additionally, as Moe and Karlsson’s review (21) included six papers in which the participants’ diagnosis was not verified or was questionable, the current review provided a more diagnostically robust synthesis of qualitative studies relating to PMDD as well as PMS. Thus, only six studies from Moe and Karlsson’s review of 12 studies were included in the current review.

To manage their enduring symptoms and maintain a functional life, women developed various maladaptive coping strategies, including disordered eating (16, 65). A strong association between suicidal ideation and PMDD was previously seen in Osborn et al.’s (20) and Prasad et al.’s (9) reviews, and reflected in the current review, in which a monthly crisis point was reached by many women. Given the level of risk highlighted, further research should focus specifically on understanding the relationship between PMDs and thoughts and attempts of suicide.

Whilst the contrast between women’s self-image with and without PMS symptoms has been documented (23), the current review noted that women’s sense of self appeared to be fractured with women describing themselves as two separate entities. Changes to identity in response to a physical health illness draw on narrative identity theory to understand the mismatch perceived identity (66). Current themes connected this fracture in identity to the guilt women felt for their behaviour during their luteal phase, and the self-objectification which followed.

Although the psychological impact PMD symptoms had on women’s quality of life has been quantified in the literature (13, 19), the current review extends these observations by recognising that even during non-symptomatic periods, women were still worrying about their next menstrual cycle. Despite the combination of PMS and PMDD diagnoses in this review, these findings were seen across all studies.

Of the eight individual sub-themes, seven were equally representative of both PMS and PMDD focused papers, highlighting that there are a number of shared experiences. However, the subtheme “taking back control” was only endorsed by papers recruiting women with PMDD. Although PMDD sits at the more severe end of the continuum, the findings of this subtheme may reflect the potentially curing treatment options for PMDD as opposed to the ongoing symptom management for women with PMS (2).

### 4.1 Strengths and limitations of synthesised papers

This review recognised the omission of relevant demographic information within the synthesised papers; 11% failed to document the participants’ age and 41% did not report their ethnicity. This omission limits the transferability of results to other groups and settings. Only five papers reported on the length of time women had experienced symptoms, or their age at onset of symptoms or diagnosis, meaning nuances within the data and psychological impact could not be explored in depth. During analysis, the authors noted that no information regarding participants’ sexuality or the gender of participants’ partners was reported in the included studies. In addition, Park et al. (48) provided no participant demographic information, and two papers did not reference the author of the chosen method of analysis. Similarly, the CASP rating scores (see **Table 3**) highlighted a notable trend of authors failing to reflect on the researcher-participant relationship (item 6), and how their own position could impact the analysis.

Whilst conducting scoping searches, the authors noted published titles which referenced a PMD; however, the methodology indicated that women without a formal diagnosis were recruited. As documented in **Figure 1**, a total of 21 papers were removed because they focused on non-clinical levels of premenstrual symptoms, and a further nine were removed due to unclear or undefined participant eligibility criteria. It is argued that this practice continues to blur the lines of what are typical premenstrual symptoms versus the severity of diagnosable PMDs. Transparency and clarity of participants’ symptoms and/or diagnosis are needed in future research.

### 4.2 Strengths and limitations of review process

This review of 17 papers was conducted in a systematic, transparent way, using an established analysis approach and synthesised the voices of 479 women across 31 years of research. Searches were independently analysed for eligibility; assessment of each paper was conducted using the validated CASP (28) checklist and initial coding was conducted separately by the two authors independently to increase credibility and minimise risk of bias. However, the decision to only include academic papers written in English raised the possibility of language, location and publication biases.

As PMS and PMDD sit within a continuum (1), studies were combined under the term PMD to develop a comprehensive picture of women’s experiences. Although it was a strength to combine qualitative PMD studies, it could also be argued that nuances of symptom severity could not be drawn out appropriately. At present PMS and PMDD are diagnosed independently; however, PMDD has only been a separate entity since 2013 (2), and hence more qualitative studies are emerging only since then. The matrix of theme representation (see **Table 4**) strengthened the decision to combine PMS and PMDD studies, as only one of eight subthemes was solely represented by both diagnoses. All seven remaining subthemes represented the experiences of women with both PMS and PMDD, highlighting the similarities of their psychological impact.

Another strength of this review was the clear specification of PMS and PMDD symptoms/diagnosis within included papers, ensuring that the synthesised data captured the experiences of women with clinical levels of symptoms, as opposed to the general population of menstruating women. Papers excluded for this reason were unlikely to represent the experiences of women with a clinically diagnosable level of symptoms. The optimum strategy to ensure formal PMS and PMDD diagnoses is debated by the research community (67). Whilst there are challenges with allowing participants to self-report their diagnosis, the validity of retrospective questionnaires has also been challenged (67). Therefore, ten papers in which participants self-reported their diagnosis were included for analysis.

### 4.3 Clinical implications

The difficult experiences women had seeking support from healthcare professionals were highlighted, adding to the concerning reality that healthcare professionals were less likely to take women’s experiences seriously (68), especially when their symptoms were related to their reproductive health (39, 69). Consequently, women with a suspected or diagnosed PMD must continue to advocate for themselves and discuss their symptoms with their family and social support network. Clinicians should consider the psychological impact of PMDs and the associated impact on quality of life, recognising the potential need for referral to clinical psychology services for therapeutic support with processing of diagnosis and psychological impact, to reduce psychological distress.

Owing to the frequency of suicidal experiences described, additional training for healthcare staff to assess PMDs and signpost women to appropriate services is required. Increased understanding of PMDs would be beneficial in healthcare services where women in crisis may present, for example, emergency services, general practitioners, and mental health teams. Once diagnosed, many women described only being offered treatment for physical symptoms. Therefore, premenstrual training for healthcare professionals is needed to have an updated understanding of the growing research into the range of evidence-based treatment options [see Nevatte et al. (70) for further exploration of treatment options] and recognise the need for therapeutic interventions targeting the psychologically distressing symptoms.

## 5 Conclusion

For the first time, qualitative papers exploring the psychological impact of premenstrual disorders (PMS and PMDD) were synthesised in one systematic review. Women described PMDs as life-changing and life-controlling, they were often left holding the responsibility for understanding and managing their own symptoms, whilst advocating for themselves in a world which did not recognise their experiences. Key recommendations included the need for medical professionals working with women in crisis, to assess for PMDs and signpost towards specialist services, including psychological interventions.

## References

[1] Ismaili E Walsh S O’Brien PMS Bäckström T Brown C Dennerstein L. Fourth consensus of the International Society for Premenstrual Disorders (ISPMD): auditable standards for diagnosis and management of premenstrual disorder. Arch Womens Ment Health. (2016) 19:953–8. doi: 10.1007/s00737-016-0631-7 27378473

[2] Goswami N Upadhyay K Briggs P Osborn E Panay N. Premenstrual disorders including premenstrual syndrome and premenstrual dysphoric disorder. Obstet Gynaecol. (2023) 25:38–46. doi: 10.1111/tog.12848

[3] Yonkers KA Casper RF. Epidemiology and pathogenesis of premenstrual syndrome and premenstrual dysphoric disorder. In: Barbieri RL Crowley WF Jr, editors. UpToDate (2018). Available at: https://www.uptodate.com/contents/epidemiology-and-pathogenesis-of-premenstrual-syndrome-and-premenstrual-dysphoric-disorder?topicRef=7382&source=see_link.

[4] The Royal College of Obstetricians and Gynaecologists. Managing premenstrual syndrome. London: RCOG. (2018). Available at: https://www.rcog.org.uk/for-the-public/browse-our-patient-information/managing-premenstrual-syndrome-pms/.

[5] Halbreich U Borenstein J Pearlstein T Kahn LS. The prevalence, impairment, impact, and burden of premenstrual dysphoric disorder (PMS/PMDD). Psychoneuroendocrinology. (2003) 28:1–23. doi: 10.1016/S0306-4530(03)00098-2 12892987

[6] Hantsoo L Epperson CN. Premenstrual dysphoric disorder: epidemiology and treatment. Curr Psychiatry Rep. (2015) 17:1–9. doi: 10.1007/s11920-015-0628-3 26377947 PMC4890701

[7] American Psychiatric Association. Diagnostic and statistical manual of mental disorders. 5th ed., text rev. Arlington VA: American Psychiatric Association (2022). doi: 10.1176/appi.books.9780890425787

[8] Santamaría M Lago I. Premenstrual experience premenstrual syndrome and dysphoric disorder. In: Psychopathology in women: Incorporating gender perspective into descriptive psychopathology. Springer International Publishing, Cham (2014). p. 423–49. doi: 10.1007/978-3-319-05870-2_18

[9] Prasad D Wollenhaupt-Aguiar B Kidd KN de Azevedo Cardoso T Frey BN. Suicidal risk in women with premenstrual syndrome and premenstrual dysphoric disorder: a systematic review and meta-analysis. J Womens Health (Larchmt). (2021) 30:1693–707. doi: 10.1089/jwh.2021.0185 PMC8721500 34415776

[10] Takeda T. Premenstrual disorders: premenstrual syndrome and premenstrual dysphoric disorder. J Obstet Gynaecol Res. (2023) 49:510–8. doi: 10.1111/jog.15484 36317488

[11] Kancheva Landolt N Ivanov K. Cognitive behavioral therapy-a primary mode for premenstrual syndrome management: systematic literature review. Psychol Health Med. (2021) 26:1282–93. doi: 10.1080/13548506.2020.1810718 32845159

[12] Carlini SV Lanza di Scalea T McNally ST Lester J Deligiannidis KM. Management of premenstrual dysphoric disorder: a scoping review. Int J Womens Health. (2022) 14:1783–801. doi: 10.2147/IJWH.S297062 PMC9790166 36575726

[13] Branecka-Woźniak D Cymbaluk-Płoska A Kurzawa R. The impact of premenstrual syndrome on women’s quality of life-a myth or a fact? Eur Rev Med Pharmacol Sci. (2022) 26:598–609. doi: 10.26355/eurrev_202201_27887 35113436

[14] Reid R. Premenstrual dysphoric disorder (formerly premenstrual syndrome). In: Feingold K Anawalt B Boyce A Chrousos G de Herder W Dhatariya K, editors. Endotext. MDText.com, Inc, South Dartmouth, MA (2000).

[15] Hardy C Hunter MS. Premenstrual symptoms and work: exploring female staff experiences and recommendations for workplaces. Int J Environ Res Public Health. (2021) 18:3647. doi: 10.3390/ijerph18073647 33807463 PMC8036722

[16] Schiola A Lowin J Lindemann M Patel R Endicott J. The burden of moderate/severe premenstrual syndrome and premenstrual dysphoric disorder in a cohort of Latin American women. Value Health. (2011) 14:93–95. doi: 10.1016/j.jval.2011.05.008 21839909

[17] Yi SJ Kim M Park I. Investigating influencing factors on premenstrual syndrome (PMS) among female college students. BMC Womens Health. (2023) 23:592. doi: 10.1186/s12905-023-02752-y 37950208 PMC10638779

[18] Prabhavathi K Kalyani-Praba P Rajendra BN Saravanan A. Cognition and psychomotor performance in premenstrual syndrome with negative emotions. BioMed Pharmacol J. (2023) 16:2061–7. doi: 10.13005/bpj

[19] Irshad A Mehmood S Noor R Mumtaz S Saleem M Laique T. Frequency of premenstrual syndrome and its association with quality of life among university students. Pak J Med Health Sci. (2022) 16:521. doi: 10.53350/pjmhs22162521

[20] Osborn E Brooks J O’Brien PS Wittkowski A. Suicidality in women with premenstrual dysphoric disorder: a systematic literature review. Arch Womens Ment Health. (2021) 24:173–84. doi: 10.1007/s00737-020-01054-8 PMC7979645 32936329

[21] Moe L Karlsson K. Is it that time of the month? Women´ s experiences of premenstrual dysphoric disorder: a review [Unpublished dissertation]. Sweden:Jonkoping University (2022).

[22] Ryan S Ussher JM Hawkey A. Mapping the abject: women’s embodied experiences of premenstrual body dissatisfaction through body-mapping. Fem Psychol. (2022) 32:199–223. doi: 10.1177/09593535211069290

[23] Cosgrove L Riddle B. Constructions of femininity and experiences of menstrual distress. Women Health. (2003) 38:37–58. doi: 10.1300/J013v38n03_04 14664304

[24] Slade P Haywood A King H. A qualitative investigation of women’s experiences of the self and others in relation to their menstrual cycle. Br J Health Psychol. (2009) 14:127–41. doi: 10.1348/135910708X304441 18442448

[25] Page MJ McKenzie JE Bossuyt PM Boutron I Hoffmann TC Mulrow CD. The PRISMA 2020 statement: an updated guideline for reporting systematic reviews. Int J Surg. (2021) 88:105906. doi: 10.1016/j.ijsu.2021.105906 33789826

[26] Cooke A Smith D Booth A. Beyond PICO: the SPIDER tool for qualitative evidence synthesis. Qual Health Res. (2012) 22:1435–43. doi: 10.1177/1049732312452938 22829486

[27] Chrisler J Gorman J. Menstruation. In: Encyclopedia of Mental Health, 3rd ed (2016). p. 75–81. doi: 10.1016/B978-0-12-397045-9.00254-8

[28] Critical Appraisal Skills Programme. CASP qualitative checklist(2018). Available online at: https://casp-uk.net/casp-tools-checklists/ (Accessed 17th November 2023).

[29] Long HA French DP Brooks JM. Optimising the value of the critical appraisal skills programme (CASP) tool for quality appraisal in qualitative evidence synthesis. Res Methods Med Health Sci. (2018) 1:31–42. doi: 10.1177/2632084320947559

[30] Harries CI Smith DM Gregg L Wittkowski A. Parenting and serious mental illness (SMI): A systematic review and metasynthesis. Clin Child Fam Psychol Rev. (2023) 26:303–42. doi: 10.1007/s10567-023-00427-6 PMC10123049 36807250

[31] Butler J Gregg L Calam R Wittkowski A. Parents’ perceptions and experiences of parenting programmes: A systematic review and meta synthesis of the qualitative literature. Clin Child Fam Psychol Rev. (2020) 23:176–204. doi: 10.1007/s10567-019-00307-y 31820298 PMC7192883

[32] Thomas J Harden A. Methods for the thematic synthesis of qualitative research in systematic reviews. BMC Med Res Methodol. (2008) 8:1–10. doi: 10.1186/1471-2288-8-45 18616818 PMC2478656

[33] Dixon-Woods M Bonas S Booth A Jones DR Miller T Sutton AJ. How can systematic reviews incorporate qualitative research? A critical perspective. Qual Res. (2006) 6:27–44. doi: 10.1177/1468794106058867

[34] Buys M. Beyond abjection: exploring narratives after premenstrual dysphoric disorder. Fem Psychol. (2024) 0:1–9. doi: 10.1177/09593535231223915

[35] Gilligan C Eddy J. The listening guide: replacing judgment with curiosity. Qual Psychol. (2021) 8:141. doi: 10.1037/qup0000213

[36] Gilligan C Spencer R Weinberg MK Bertsch T. On the Listening Guide: A voice-centered relational method. In: Camic PM Rhodes JE Yardley L, editors. Qualitative research in psychology: Expanding perspectives in methodology and design. American Psychological, Washington, DC (2003). p. 157–72.

[37] Chan K Rubtsova AA Clark CJ. Exploring diagnosis and treatment of premenstrual dysphoric disorder in the U.S. healthcare system: a qualitative investigation. BMC Womens Health. (2023) 23:272. doi: 10.1186/s12905-023-02334-y 37198676 PMC10193729

[38] Campbell R Wasco SM. Feminist approaches to social science: epistemological and methodological tenets. Am J Community Psychol. (2000) 28:773–91. doi: 10.1023/A:1005159716099 11109478

[39] Osborn E Wittkowski A Brooks J Briggs PE O’Brien PS. Women’s experiences of receiving a diagnosis of premenstrual dysphoric disorder: a qualitative investigation. BMC Womens Health. (2020) 20:1–15. doi: 10.1186/s12905-020-01100-8 33115437 PMC7594422

[40] Steiner M Macdougall M Brown E. The premenstrual symptoms screening tool (PSST) for clinicians. Arch Womens Ment Health. (2003) 6:203–9. doi: 10.1007/s00737-003-0018-4 12920618

[41] Braun V Clarke V. Using thematic analysis in psychology. Qual Res Psychol. (2006) 3:77–101. doi: 10.1191/1478088706qp063oa

[42] Braun V Clarke V. Reflecting on reflexive thematic analysis. Qual Res Sport Exerc Health. (2019) 11:589–97. doi: 10.1080/2159676X.2019.1628806

[43] Marfuah D Barat N. Premenstrual dysphoric disorder causes discomfort and interfere adolescent’s social relationship. Indones J Nurs Midwifery. (2018) 6:219–25. doi: 10.21927/jnki.2018.6(3

[44] Hardy C Hardie J. Exploring premenstrual dysphoric disorder (PMDD) in the work context: a qualitative study. J Psychosom Obstet Gynaecol. (2017) 38:292–300. doi: 10.1080/0167482X.2017.1286473 28635534

[45] Jurvanen SH. The subjective experience of premenstrual dysphoric disorder (PMDD): a qualitative study exploring consequences of PMDD symptoms in relation to occupational and private life [Unpublished bachelor’s thesis]. Sweden: Lund University (2017).

[46] Labots-Vogelesang MS Kooiman-Andringa R Teunissen TAM Lagro-Janssen ALM. Perspectives of Dutch women on premenstrual disorder: a qualitative study exploring women’s experiences. Eur J Gen Pract. (2023) 29:2166033. doi: 10.1080/13814788.2023.2166033 36714999 PMC9888467

[47] Malterud K. Systematic text condensation: a strategy for qualitative analysis. Scand J Public Health. (2012) 40:795–805. doi: 10.1177/1403494812465030 23221918

[48] Park Y Murphy A Cezar da Cruz D. Occupational participation and engagement of woman experiencing premenstrual syndrome: a qualitative study. Br J Occup Ther. (2023) 86:639–47. doi: 10.1177/03080226231174792 PMC12033791 40336715

[49] Tutty LM Barry L Nixon KL. Mommy’s having a bad day”: the impact of premenstrual symptoms on mothering. Womens Reprod Health. (2022) 9:81–99. doi: 10.1080/23293691.2021.2016137

[50] Ussher JM Perz J. I feel fat and ugly and hate myself”: self-objectification through negative constructions of premenstrual embodiment. Fem Psychol. (2020) 30:185–205. doi: 10.1177/0959353519900196

[51] Ussher JM Perz J. Evaluation of the relative efficacy of a couple cognitive-behaviour therapy (CBT) for premenstrual disorders (PMDs), in comparison to one-to-one CBT and a wait list control: a randomized controlled trial. PloS One. (2017) 12:1–25. doi: 10.1371/journal.pone.0175068 PMC5395168 28419170

[52] Labots-Vogelesang MS Andringa RK Teunissen DA Janssen ALL. A women’s perspective on premenstrual syndrome: a qualitative interview study [Manuscript submitted for publication]. (2019). doi: 10.21203/rs.2.10868/v1

[53] Booth A Hannes K Harden A Noyes J Harris J Moher D. Consolidated Criteria for Reporting Qualitative Studies COREQ. Guidelines for reporting health research: a user’s manual. Oxford: John Wiley & Sons (2014).

[54] Siahbazi S Montazeri A Taghizadeh Z Masoomie R. The consequences of premenstrual syndrome on the quality of life from the perspective of affected women: a qualitative study. J Res Med Dent Sci. (2018) 6:284–92. doi: 10.5455/jrmds.20186243

[55] Polit DF Tatano BC. Nursing Research: Generating and Assessing Evidence for Nursing Practice. 8th ed. Philadelphia: Lippincott Williams & Wilkins (2008).

[56] Ussher JM Perz J. PMS as a process of negotiation: women’s experience and management of premenstrual distress. Psychol Health. (2013) 28:909–27. doi: 10.1080/08870446.2013.765004 23383644

[57] Ussher JM. Premenstrual syndrome: reconciling disciplinary divides through the adoption of a material-discursive-intrapsychic approach. In: Kolk A Bekker M Van Vliet K, editors. Advances in Women and Health Research. Tilberg University Press, Amsterdam (1999). p. 47–64.

[58] Hoga LAK Vulcano MA Miranda CM Manganiello A. Male behaviour in front of women with premenstrual syndrome: narratives of women. Acta Paul Enferm. (2010) 23:372–8. doi: 10.1590/S0103-21002010000300010

[59] Reissman CK. Narrative Analysis. London: Sage (1993).

[60] Mooney-Somers J Perz J Ussher JM. A complex negotiation: women’s experiences of naming and not naming premenstrual distress in couple relationships [corrected]. Women Health. (2008) 47:57–77. doi: 10.1080/03630240802134134 18714712

[61] Stenner P. Discoursing jealousy. In: Burman E Parker I, editors. Discourse Analytic Research: Repertoires and Readings of Texts in Action. Routledge, London (1993). p. 94–132.

[62] Perz J Ussher JM. Women’s experience of premenstrual syndrome: a case of silencing the self. J Reprod Infant Psychol. (2006) 24:289–303. doi: 10.1080/02646830600973883

[63] Burrage J Schomer H. The premenstrual syndrome: perceived stress and coping efficacy, physical & somatic disorders. S Afr J Psychol. (1993) 23:111–5. doi: 10.1177/008124639302300302

[64] Stone PT Dunphy DC Smith MS Ogilvie DM. The General Enquirer: A Computer Approach to Content Analysis. Cambridge, Massachusetts: MIT Press (1966).

[65] Coban OG Karakaya D Onder A Isleyen Z Adanir AS. Association of premenstrual dysphoric disorder and eating behaviors among nursing students: a cross-sectional study. J Pediatr Adolesc Gynecol. (2021) 34:203–8. doi: 10.1016/j.jpag.2020.11.019 33271293

[66] Walker MJ Rogers WA. Diagnosis, narrative identity, and asymptomatic disease. Theor Med Bioeth. (2017) 38:307–21. doi: 10.1007/s11017-017-9412-1 28681328

[67] Eisenlohr-Moul T. Premenstrual disorders: a primer and research agenda for psychologists. Clin Psychol. (2019) 72:5. doi: 10.31234/osf.io/tw4bd 32362679 PMC7193982

[68] Hoffmann DE Tarzian AJ. The girl who cried pain: a bias against women in the treatment of pain. J Law Med Ethics. (2001) 29:13–27. doi: 10.1111/j.1748-720X.2001.tb00037.x 11521267

[69] Grundström H Alehagen S Kjølhede P Berterö C. The double-edged experience of healthcare encounters among women with endometriosis: a qualitative study. J Clin Nurs. (2018) 27:205–11. doi: 10.1111/jocn.13872 28493635

[70] Nevatte T O’Brien PMS Bäckström T Brown C Dennerstein L Endicott J. ISPMD consensus on the management of premenstrual disorders. Arch Womens Ment Health. (2013) 16:279–91. doi: 10.1007/s00737-013-0346-y PMC3955202 23624686

